# Integrated clinical analysis and data mining assessed the impact of NOX4 on the immune microenvironment and prognosis of pancreatic cancer

**DOI:** 10.3389/fonc.2023.1044526

**Published:** 2023-02-16

**Authors:** Xin Zhao, Yichen He, Yi Pan, Luyi Ye, Longcai Liu, Xiaozhou Mou, Luoqin Fu

**Affiliations:** ^1^ General Surgery, Cancer Center, Department of Hepatobiliary & Pancreatic Surgery and Minimally Invasive Surgery, Zhejiang Provincial People’s Hospital, Affiliated People’s Hospital, Hangzhou Medical College, Hangzhou, Zhejiang, China; ^2^ Department of Pharmacy, Hangzhou Medical College, Hangzhou, Zhejiang, China; ^3^ College of pharmacy, Zhejiang University of Technology, Hangzhou, Zhejiang, China

**Keywords:** pancreatic cancer, NOX4, lncRNAs, infiltrating immune cells, immune subtype, prognostic model, AC092667.2, RP11-349A8.3

## Abstract

**Background:**

The tumor microenvironment (TME) of pancreatic cancer is complex. which forms forms a microenvironment with high immunosuppression, ischemia and hypoxia, which promotes tumor proliferation and migration, inhibit the anti-tumor immune response. NOX4 plays an important role in tumor microenvironment and has a significant relationship with the occurrence, development and drug resistance of tumor.

**Methods:**

Firstly, NOX4 expression in pancreatic cancer tissues under different pathological conditions was detected by applying immunohistochemical staining of tissue microarray (TMA). Transcriptome RNA sequencing data and clinical data of 182 pancreatic cancer samples were downloaded and collated from the UCSC xena database. 986 NOX4-related lncRNAs were filtered by Spearman correlation analysis. prognosis-related NOX4-related lncRNAs and NRlncSig Score were finally obtained by univariate and multivariate Cox regression with Least Absolute Shrinkage and Selection Operator (Lasso) analysis in pancreatic cancer patients. we plotted Kaplan -Meier and time-dependent ROC curves (ROC) to assess the validity in predicting the prognosis of pancreatic cancer. The ssGSEA analysis was applied to explore the immune microenvironment of pancreatic cancer patients as well as to discuss the immune cells and immune status separately.

**Results:**

We found that a mature tumor marker, NOX4, play different roles in different clinical subgroups by immunohistochemical analysis and clinical data. Finally, 2 NOX4-related lncRNAs were determined by least absolute shrinkage and selection operator (LASSO) analysis, univariate Cox analysis and multivariate COX analysis. The ROC curve and DCA curve showed that NRS Score had better predictive ability than independent prognosis-related lncRNA and other clinicopathologic indicators. We obtained the relative abundance of 28 infiltrating immune cells by ssGSEA analysis and found a significant positive correlation between the abundance of anti-tumor immune cells and tumor-promoting immune cells in the risk-classified microenvironment. No matter NRS Score or AC092667.2, RP11-349A8.3 was significantly correlated with immune infiltrating cells. Meanwhile, the IC50 of conventional chemotherapeutic agents in high-score group were significantly lower than those in low-score group.

**Conclusion:**

As a mature tumor marker, NOX4-related lncRNAs provide new research strategies for prognostic evaluation, molecular mechanism and clinical treatment of pancreatic cancer.

## 1 Introduction

Pancreatic cancer is a malignant tumor of the digestive tract with insidious onset, rapid progression, and poor prognosis. In recent years, its morbidity and mortality in the global scope are increasing year by year. The latest statistics from the China National Cancer Center show that the incidence of pancreatic cancer has risen to the ninth place and the death rate is in the sixth place, its share of cancer related deaths has increased by 9% over the past decade (1). The tumor microenvironment (TME) of pancreatic cancer is complex. The cellular and non-cellular components in TME interact with each other and form a microenvironment with high immunosuppression, ischemia and hypoxia, which promotes tumor proliferation and migration, inhibit the anti-tumor immune response (2–4). On the one hand, the abundant stroma makes it difficult for drugs to penetrate the tumour tissue, and establish an immunosuppressive tumor microenvironment. In recent years, immunotherapy has had significant effects on many malignant tumors, but these effects have not been shown satisfactorily in pancreatic cancer (5). The main causes are poor effector T cell infiltration and prominent myeloid inflammation typical of immunosuppressive tumor microenvironments (6–8), as well as a low mutation burden producing minimal immunogenic antigens (9, 10). However, a small number of cancer patients showed high effector T cell infiltration and long overall survival, suggesting the potential for effective immunotherapy for pancreatic cancer (11–13). Therefore, it is urgent to study the pathological mechanism of pancreatic cancer and to find early detection markers to improve the survival time and prognosis of patients with pancreatic cancer.

The human NADPH oxidase 4 (NOX4) gene is located on chromosome 11q14.2-q21, with a minimum length of 265kb (14). In most tumor types, NOX4 significantly promotes tumor development (15). NOX4 interacts with IL-6/STAT3 and PI3K/Akt signaling pathways to promote non-small-cell lung carcinoma cell proliferation and survival (16). Studies have found that NOX4 supports apoptosis of malignant pancreatic cancer cells by increasing Reactive oxygen species (ROS) levels and DNA damage (17). During the invasion of pancreatic cancer, NOX4 induces epithelial-mesenchymal transformation through Rac/p38 MAPK related pathway, and up-regulates E-cadherin through transforming growth factor-β pathway to promote cell migration (18). NOX4 is involved in almost every process of the occurrence, development and metastasis of cancers. NOX4 may also promote the resistance of cancer cells to chemotherapeutic agents and radiotherapy (19). At the same time, NOX4, as a ROS producing enzyme, is the downstream target of TGF-β1 and the regulatory center of cancer-associated fibroblast phenotype in many cancers, and promotes cancer-associated fibroblast (CAF) activation in tumors, with strong CAF specificity (20). NOX4 is involved in the development of pancreatic cancer in various forms, mediating the activation of signaling molecules in multiple pathways and altering cellular activity in the tumor microenvironment. This makes it feasible to advance drug therapy or gene therapy in clinical trials with NOX4 as the primary target.

However, throughout the complex environment of tumor development, abnormal gene expression is implicated in protein-protein interactions and a series of cascade reactions are activated. In turn, long non-coding RNAs (lncRNAs) are not only involved in epigenetic modifications but also interfere with protein translation. LncRNAs can induce many phenotypes of tumor cells through interaction with other biological molecules. Some cancer-related lncRNAs have been identified as potential cancer therapy targets. PGEM1, a highly tissue-specific lncRNA, has been found to be highly expressed in prostate cancer. It can delay the induction of p53 and p21, inhibit the division of PARP and inhibit the apoptosis induced by adriamycin, and then promote the chemotherapy resistance (21). The expression of lncRNA has also been shown to be associated with immune responses and tumor progression, which may play a key regulatory role in the innate immune system and adaptive immune system. lncRNAs can form regulatory complexes with miRNA, mRNA or protein to regulate the expression of immune cells in the microenvironment and the expression of genes in these immune cells. lncRNAs were found to be involved in the modulation of macrophage polarization towards M1/M2 phenotype and the alteration of cellular signaling pathways. LncRNA RP11-361F15.2 can positively regulate the M2 macrophage polarization of tumor-associated macrophages by interacting with miR-30c-5p/CPEB4 (22). LncRNA MAF4 can induce T helper 1 cell development and inhibit Th2 differentiation, which leads to imbalance of Th1/Th2 cells (23). Supported by these studies, it is necessary to explore survival-specific lncRNAs in new subtypes of pancreatic cancer. At the same time, the regulation of these novel nucleic acid molecules in the immune microenvironment is also worth exploring. Unfortunately, studies focusing on the relationship between NOX4 and lncRNAs in tumors have been neglected. While, the results of studies for brain injury and diabetic nephropathy demonstrate that lncRNAs play an important role in the NOX4-related signaling axis. Exploring the role played by NOX4-related lncRNAs in pancreatic cancer is pioneering and meaningful.

Improving the accuracy and sensitivity of early screening for pancreatic cancer is still the best way to improve the survival rate of patients with pancreatic cancer, and finding new tumor markers with clinical value plays an important role in its diagnosis. At this stage, the research results on the function of NOX4 in cellular metabolism and the signal transduction in the tumor microenvironment are widely recognized, at the cellular level and in mouse tumor models. This proves that NOX4 can be more quickly applied as a marker for the diagnosis and treatment of pancreatic cancer on clinical trials. However, the status of real cases is often beyond expectation and more complex, and is difficult to match with the ideal situation in the research models. To avoid the potential risk of preclinical application, more patient-based studies need to be carried forward. Analysis of pathological samples from a large number of pancreatic cancer patients seems to be the most ideal and ethical. At the same time, we found that the relationship between NOX4 and lncRNAs in pancreatic cancer has rarely been studied, and the functions of NOX4-related lncRNAs in the tumor microenvironment and immune microenvironment have rarely been explored. Based on the above clinical problems, we discussed the NOX4-related lncRNAs in pancreatic cancer, with NOX4 as the characteristic factors. In this study, the expression of NOX4 in pancreatic cancer tissues was detected by immunohistochemical staining of tissue microarrays, and their effects were confirmed. To increase the understanding of the role of NOX4 in the pathologic process of pancreatic cancer based on a large number of clinicopathologic samples. And combined with the public database, the expression of NOX4-related lncRNAs in human pancreatic cancer was analyzed to explore their relationship with prognosis and immune microenvironment of patients with pancreatic cancer, immune cells and immune status were explored respectively. This may provide an opportunity for the development of immunotherapy and targeted therapy for pancreatic cancer in the future.

## 2 Materials and methods

### 2.1 Tissue samples and construction of tissue microarray

The 362 paraffin specimens of pancreatic cancer were acquired to construct tissue microarrays (TMA) form the Department of Surgery, Zhejiang Provincial People’s Hospital, having been collected from 2012 to 2017. Based on the clinical data collected, 205 pancreatic cancer samples were reliably annotated for subsequent prognostic analysis. All cases were classified according to the World Health Organization classification criteria of tumor pathology. Before surgical interventions, none of the patients received any chemotherapy. All tissue samples were taken with the patient’s informed consent during surgical procedures. The tissue sample was fixed in formalin and then embedded in paraffin.

The project has been approved by the Ethics Committee of Zhejiang Provincial People’s Hospital.

### 2.2 Immunohistochemistry analysis

Avidin-biotin-pcroxidase complex method was used for immunohistochemical analysis of 4-μm-thick tissue sections fixed in formalin and embedded in paraffin. The standard procedures for xylene and graded alcohol were used to deparaffinized and dehydrated the TMA sections to ensure optimal treatment conditions for each antibody. NOX4 immunostained sections were pretreated at high voltage for 3 minutes in 0.01M sodium citrate antigenic retrieval buffer (pH 6.0). The sections are then cooled down to room temperature for 20 minutes. The activity of endogenous peroxidase was blocked with 3% H_2_O_2_. Sections were incubated with 10% nonimmune goat serum at room temperature for 30 minutes to reduce non-specific binding in background. NOX4 immunostained sections were incubated overnight with primary antibody (1:500 dilution, ABCAM, USA) at 4°C. The sections were then incubated at room temperature for 20 minutes with a biotin-labeled second antibody (Thermo Fisher Scientific, Inc., Waltham, MA, USA). After rinsing with phosphate-buffer saline, the sections were incubated at room temperature for 20 minutes with HRP-Conjugated Streptavidin (Invitrogen; Thermo Fisher Scientific, USA). The tissue sections were stained with 3,3-diaminobenzidine, lightly counterstained with hematoxylin and, dehydrated and mounted.

A digital tissue section scanner or imaging system (Pannoramic MIDI, 3DHISTECH, Hungary) was used to capture the image or scan file on the immunohistochemical section, and an image analysis system was used to read the tissue measurement area automatically. First, the positive grades were classified: 1, weak positive, light yellow; 2, moderate positive, brownish-yellow; 3, strong positive, sepia. Then the weak, moderate and strong positive areas were calculated. The area of tissues, the positive cumulative optical density (IOD) value and the positive area were calculated. The positive area ratio, mean optical density and positive area density were calculated respectively to reflect the positive degree. The positive intensity was evaluated by Histochemistry score.

Positive area ratio: positive area/tissue area. Reflect the positive area.

Mean optical density: positive IOD value/positive area. The average depth of the positive signal.

Positive area density: positive IOD value/tissue area. Reflect the average depth of positive in the whole measuring area.

H-score: Histochemistry score is a histological scoring method for immunohistochemical staining. The number of positive cells in each section and its staining intensity are converted into corresponding values for semi-quantitative analysis of histochemistry. H-Score (H-Score = ∑ (pi × i) = (percentage of weak intensity area × 1) + (percentage of moderate intensity area × 2) + (percentage of strong intensity area × 3). The H-score was between 0 and 300. The larger the score, the stronger the overall positive intensity.

### 2.3 Clinical samples and data acquisition

Transcriptome RNA sequencing data from 183 pancreatic cancer samples were downloaded from the UCSC xena database (https://xena.ucsc.edu/). Meanwhile, Clinical data of patients with pancreatic cancer were also obtained, such as sex, age, tumor stage, total survival time, survival status, etc. the GeneCards database (https://www.GeneCards.org/). Human genome annotation GTF file are available in the Ensemble database (https://uswest.ensembl.org/) and Gencode database (https://www.gencodegenes.org/).

### 2.4 Differential gene analysis and clustering analysis

After sorting and filtering the gene expression profiling data, the difference was analyzed using “RUVseq” algorithm and “DEseq2” algorithm of R software. We set the filter threshold to log_2_ | fold change | > 1 and *p*< 0.05, to obtain differentially expressed genes for subsequent analysis and to annotate differentially expressed genes (DEGs) associated with NOX4. The correlation between NOX4-related differentially expressed genes and lncRNAs was calculated by test function pair, and the correlation coefficient | r^2^ | > 0.3 and *p* < 0.05 were used as screening criteria. Using the “org.HS.Eg.db” and “Clusterprofiler” packages of R software, the GO and KEGG enrichment of NOX4-related differential genes were analyzed.

### 2.5 Construction of prognosis analysis forNOX4-Related lncRNAs

First, LASSO regression model was used to screen NOX4-related lncRNAs, and 10-fold cross-validation was used, the gene whose regression coefficient is not penalized to 0 is selected as the feature elements (*p value* with a threshold value of 0.05). We used univariate Cox analysis for the NOX4-related lncRNAs associated with prognosis in patients with pancreatic cancer and multivariate Cox regression analysis for the NOX4-related lncRNAs associated with prognosis. NOX4-related lncRNAs with independent predictive value were incorporated into the model construction and established risk scores.

NRlncSig Score (NRS Score) = $\sum_{i-1}^{n}e^{coef(\ln cRNA)}*expr(lncRNA)$ coef (lncRNA) and expr (lncRNA) represent survival-related regression coefficients and expression values of NOX4-related lncRNAs.

Combining with clinicopathologic features, a nomogram was established to predict 1-, 3-and 5-year survival rates in patients with pancreatic cancer. At the same time, in order to study the sensitivity of patients with pancreatic cancer to different chemotherapeutic drugs, the “oncoPredict” package of R software was used to predict the half maximal inhibitory concentration (IC50) of pancreatic cancer-related chemotherapeutic drugs.

### 2.6 Immune infiltration in different patients was verified by single sample gene set enrichment analysis

We downloaded and filtered the RNAseq expression profile data of PAAD. 28 types of immune infiltrating cells in each patient were annotated with the R “GSVA” package, and their abundance was analyzed to determine immune infiltrating status in an independent sample. The expression profiles of immune cells were standardized for subsequent analysis.

### 2.7 Relationship between immune score and prognosis-related lncRNAs

The expression matrix of RNA-seq in pancreatic cancer was analyzed, and the stromal cells and immune cells in tumor tissue were evaluated by the “ESTIMATE” algorithm of R software, the level of stromal and the infiltration degree of immune cells in tumor tissues were measured to explore the immune status under different risk levels. In addition, the correlation between immune score and prognosis-related lncRNAs was analyzed.

### 2.8 Identification of immune subtypes

Using the R package “ConsensusClusterPlus”, we performed an unsupervised consensus clustering for the immune cell expression profile of PAAD patients with 1,000 iterations based on k-means machine learning algorithm, each iteration sampled 80% of the data. According to relative change in area under CDF curve, patients were classified into different immune subtypes.

### 2.9 Gene set enrichment analysis

Gene Set Enrichment Analysis (GSEA) was performed in patients with high score and low score by R software. GSEA was carried out between immune subtype cluster 1 and immune subtype cluster 2 to explore the statistical differences in KEGG pathway enrichment. The gene set “c2.cp.kegg.v7.5.1. ENTREZ.GMT” were selected as the reference gene set.

### 2.10 Statistical analysis

The samples used for the survival analysis contained complete clinical information and were analyzed using the “survival” package. The survival probability was calculated by Kaplan-Meier method. The “corrplot” package of R software is used to analyze the correlation. Determining the normal distribution of data by using the Shapiro-Wilk test. The statistical significance of the difference between the normally distributed variables was estimated by the student t-test, and the non- normally distributed variables were analyzed by the Mann-Whitney U test. Spearman correlation analysis was used in correlation calculation. Univariate and multivariate COX proportional hazard regression models adjusted or unadjusted for available prognostic clinical covariates were used to calculate hazard ratios (HR) and 95% confidence intervals by the R packages “Survminer” and “Survival”. The specificity and sensitivity of survival model were evaluated by time-dependent ROC curve and DCA decision curve. All statistical analyses were performed using R software (version 4.1.2). *p* < 0.05 was considered as statistically significant.

## 3 Results

### 3.1 Expression of NOX4 in pancreatic tumors

**Figure 1:**
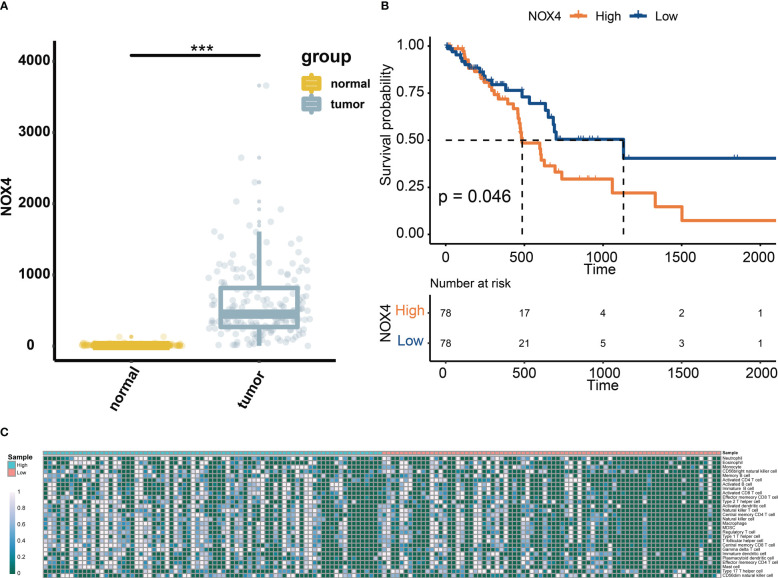
Expression of NOX4 in pancreatic tissue. **(A)** The levels of NOX4 expression in pancreatic tumors and normal tissues from TCGA and GTEx databases. **(B)** Kaplan–Meier curve for patients with high or low expression of NOX4. **(C)** Abundance of immune cells in pancreatic cancer patients with high and low expression of NOX4. ***p < 0.001.

**Figure 2:**
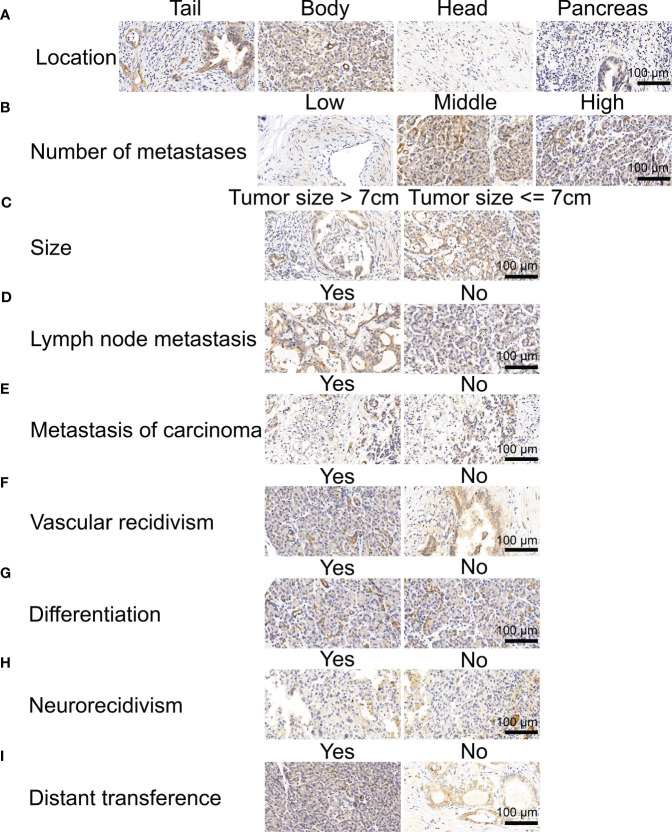
Immunohistochemical analysis of NOX4 in pancreatic cancer. **(A)** Immunohistochemical analysis of patients in different location. **(B)** Immunohistochemical analysis of patients with different numbe of metastases. **(C)** Immunohistochemical analysis of patients with different tumor size. **(D)** Immunohistochemical analysis of patients with and without lymph node metastasis. **(E)** Immunohistochemical analysis of patients with and without metastasis of carcinoma. **(F)** Immunohistochemical analysis of patients with and without vascular recidivism. **(G)** Immunohistochemical analysis of patients with and without differentiation. **(H)** Immunohistochemical analysis of patients with and without neurorecidivism. **(I)** Immunohistochemical analysis of patients with and without distant metastasis.

We first analyzed the levels of NOX4 expression in pancreatic tumors and normal tissues from TCGA and GTEx databases, and found that NOX4 was overexpressed in the tumor tissues (**Figure 1A**). Patients with high expression of NOX4 were associated with poor prognosis (**Figure 1B**). Moreover, the differential NOX4 expression in pancreatic cancer patients seems to affect the expression of immune cells (**Figure 1C**). The immunostaining of NOX4 were mainly located in cell membrane and cytoplasm. We found the highest expression of NOX4 in pancreatic body tumors, and in patients with Tumor Size less than 7 cm, with moderate lymph node metastasis, no vascular recidivism or no distant metastasis, high expression of NOX4 was detected in the pancreas (**Figures 2A-D, F, I**). And no matter whether with tumor metastasis, differentiation or neurorecidivism, didn’t seem to affect NOX4 expression in tumor (**Figures 2E, G, H**).

### 3.2 Survival analysis of patients with pancreatic cancer

**Figure 3:**
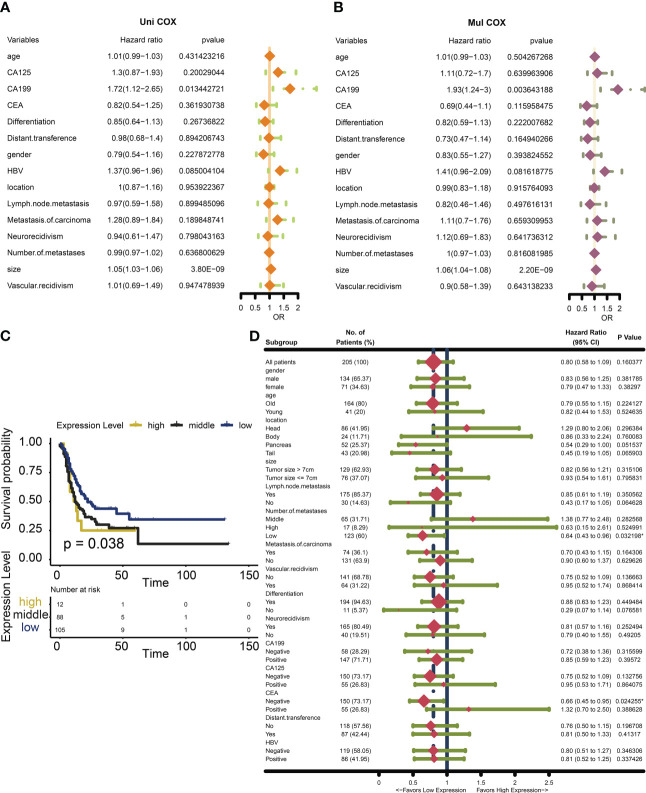
Survival analysis and Subgroup analysis of NOX4 in clinicopathologic features of pancreatic cancer. **(A)** Univariate Cox analysis of clinicopathologic features in patients with pancreatic cancer. **(B)** Multivariate Cox analysis of clinicopathologic features in patients with pancreatic cancer. **(C)** Kaplan-Meier curves of different NOX4 expression levels. **(D)** Subgroup analysis of NOX4 expression.

We found that in addition to tumor size, common clinical features do not seem to be a better assessment of patient survival (**Figures 3A, B**). We found that low expression of Nox4 seems to be a marker of good prognosis (**Figure 3C**). H-Score was used to evaluate the effect of NOX4 on the survival rate of patients with pancreatic cancer in each clinical characteristic subgroup. The results suggest that NOX4 appears to have an important effect on survival in patients with low lymph node metastases and in patients with carcinoembryonic antigen (CEA) negative (**Figure 3D**).

### 3.3 Functional analysis of differentially expressed genes related to NOX4

**Figure 4:**
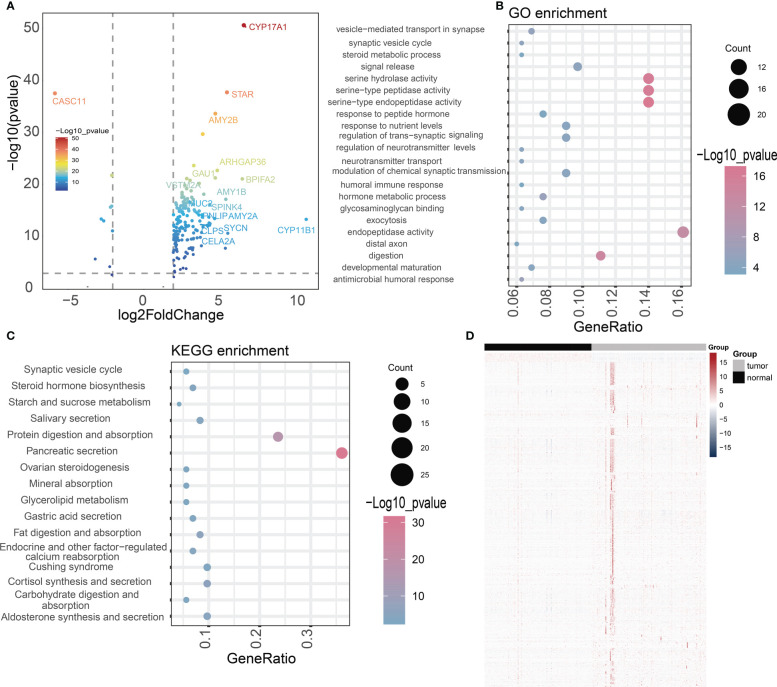
Difference analysis and enrichment analysis. **(A)** The volcano map shows NOX4-related differential expression genes. **(B, C)** GO and KEGG analyses for NOX4-related differentially expressed genes. **(D)** Heatmap of the lncRNAs highly correlated with NOX4 in pancreatic tumors and normal tissues.

To better understand the relationship of NOX4 gene expression in pancreatic cancer, we downloaded RNA-seq data from the TCGA database of 182 pancreatic cancer patients, the patients were divided into high-expression group and low-expression group according to the median expression of NOX4 genes. The 168 differentially expressed genes (DEGs) were identified by NOX4 classification, including 156 up-regulated genes and 10 down-regulated genes (**Figure 4A**). GO analysis of these common DEGs showed that most of them were enriched to ndopeptidase activity, serine hydrolase activity and response to nutrient levels (**Figure 4B**). The enrichment of KEGG pathway indicates that Pancreatic secretion, Protein digestion and absorption, Salivary secretion and Steroid hormone biosynthesis are enriched (**Figure 4C**).

### 3.4 Identification and prognostic analysis of NOX4-related lncRNAs

**Figure 5:**
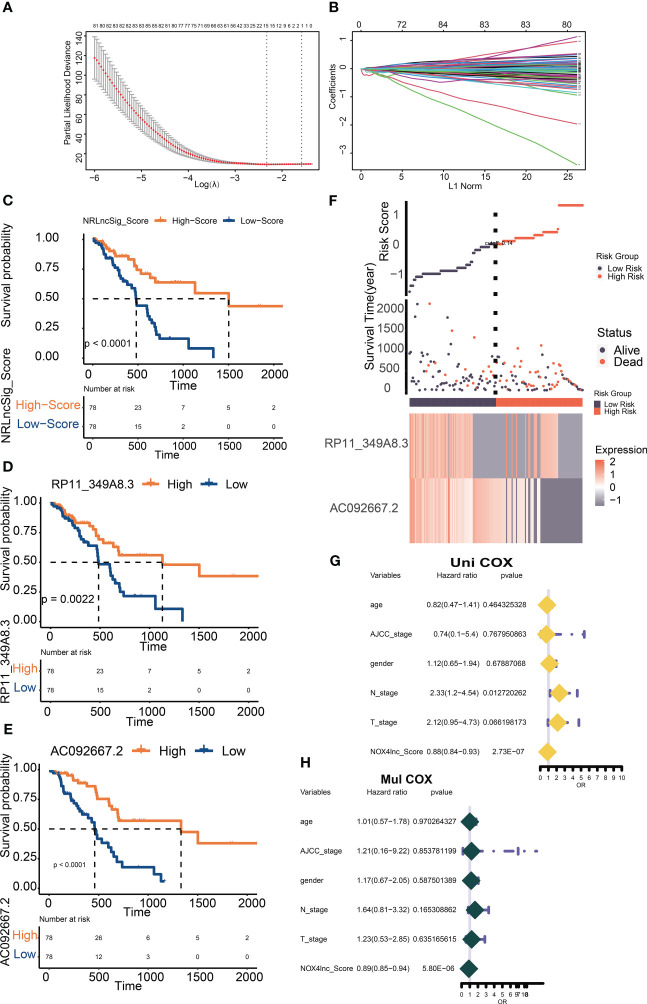
Screening and verification of prognostic NOX4-related lncRNAs. **(A)** Plots for LASSO expression coefficients of NOX4-related lncRNAs. **(B)** Cross-validation plot for the penalty term. **(C)** Kaplan-Meier evaluated the overall survival of the low-score or high-score patients predicted by NRS Score. **(D, E)** Kaplan–Meier survival curve of overall survival based on the expression of RP11-349A8.3, AC092667.2. **(F)** Rank, distribution, and median values obtained based on risk scores; the survival status and the expression of lncRNA in different groups of patients with pancreatic cancer. **(G, H)** Cox-regression analysis of NRS Score and clinicopathologic features.

DEGs based on NOX4 expression does not seem to adequately explain the important role of NOX4 in the tumor microenvironment of pancreatic cancer. Through Spearman rank correlation analysis, we select lncRNAs with correlation coefficient > 0.9 for the exploration of follow-up mechanism. Eventually, 986 NOX4-related lncRNAs were screened out (**Figure 4D**). The genes were then entered into the LASSO regression model and 12 genes were screened as feature vectors under penalty conditions (α = 1) (**Figures 5A, B**). Through univariate and multivariate COX regression analysis of these lncRNAs, 2 NOX4-related lncRNAs were identified as having important prognostic significance (**Figure 5C**), it was used to construct the prognostic signature: NRlncSig Score (NRS Score) = (0.864 × AC092667.2 expression) + (0.915 × RP11-349A8.3 expression). Kaplan-Meier survival analysis showed that high- score was significantly associated with better prognosis (p < 0.0001), and survival time was significantly shorter in the low-score group (**Figure 5C**). The NRS Score of each patient was calculated according to the formula, and the pancreatic cancer samples were divided into high-score group and low-score group according to the median of the NRS Score. With the increase of the score, the number of deaths decreased significantly and the overall survival time was longer. The heatmap shows that the expression of AC092667.2, RP11-349A8.3 were higher in low-score group (**Figure 5F**). At the same time, we found a significant association between high expression of these two lncRNAs and good prognosis (**Figures 5D, E**). These results were also verified by univariate and multivariate Cox regression analysis (**Figures 5G, H**).

### 3.5 Evaluation and verification of prognostic signature

**Figure 6:**
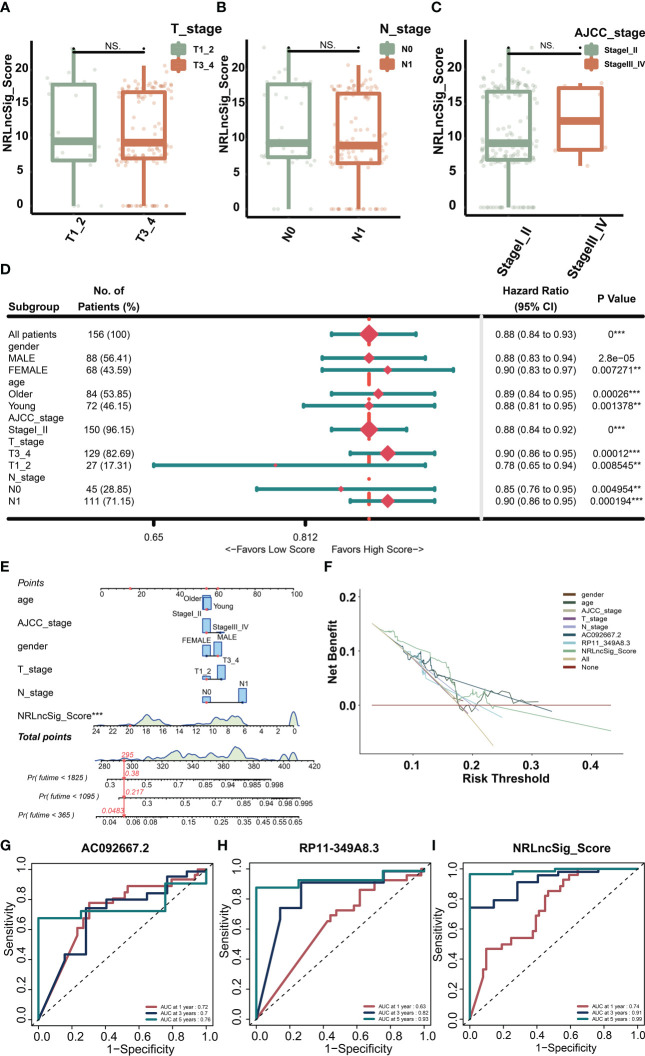
Validation of the NRS score model. **(A)** NRS Score in patients with different T stages. **(B)** NRS Score in patients with different N stages. **(C)** NRS Score in patients with different AJCC stages. **(D)** Clinical subgroup analysis of NRS Score. **(E)** Nomogram integrating clinicopathologic features and NRS Score. **(F)** The DCA curve of NRS Score with clinicopathologic features. **(G–I)** The time-dependent ROC curve of RP11-349A8.3, AC092667.2 and NRS Score. **p < 0.01, ***p < 0.001, NS indicates no significant difference.

We screened common clinical staging indicators and found that NRS Score did not differ significantly among patients with different T, N or AJCC stage (**Figures 6A-C**). In order to better explain the predictive value of NRS Score based on NOX4 stratification, we performed a subgroup analysis based on NRS Score. The results showed that NRS Score was a very sensitive predictor of survival in most subgroup patients with pancreatic cancer (**Figure 6D**). The time-dependent ROC curve also showed the predictive ability of AC092667.2, RP11-349A8.3 and NRS Score for patient survival. It can be seen that Signature is ideal for the evaluation of 1 -, 3 -, and 5-year survival rates of patients. The area under curve (AUC) was 0.74, 0.91 and 0.99 (**Figures 6G-I**). Compared with NOX4-related lncRNAs and clinical indicators, Signature’s Decision Curve Analysis (DCA) also showed a good net benefit (**Figure 6F**). Nomogram combined with clinicopathologic features and NRS Score is stable and accurate, and can be used to evaluate the prognosis of pancreatic cancer (**Figure 6E**).

### 3.6 Evaluation of immune infiltration

**Figure 7:**
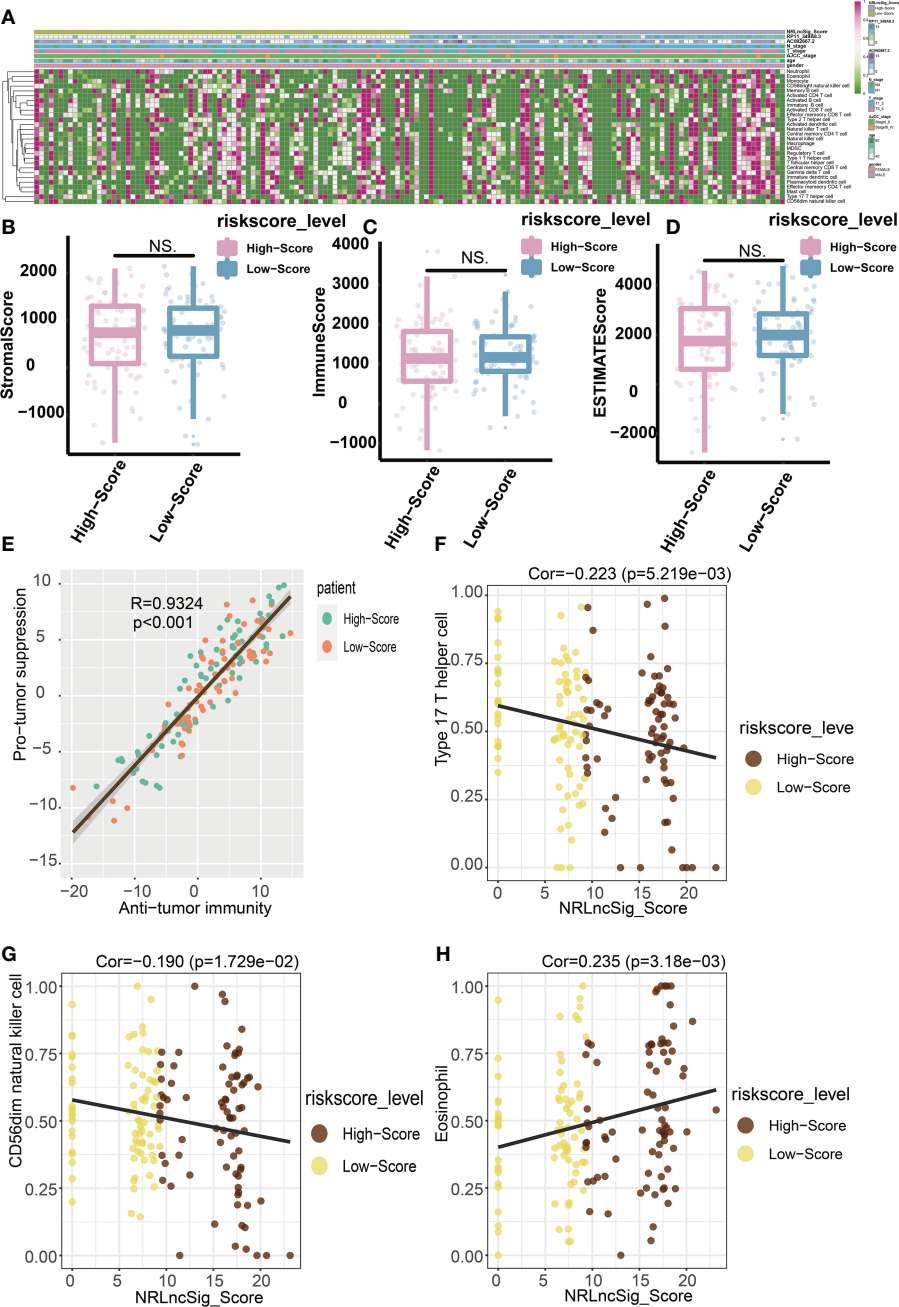
Predictive value of NRS Score for immunotherapy. **(A)** Relative abundance of 28 infiltrating immune cells. **(B)** StromalScore of high-score group and low-score group. **(C)** ImmuneScore of high-score group and low-score group. **(D)** ESTIMATEScore of high-score group and low-score group. **(E)** The abundance of immune cells with anti-tumor function and tumor-suppressor immune cells was significantly positively correlated in microenvironment classified by risk score. **(F)** Correlation analysis between NRS Score and Type 17 T helper cell. **(G)** Correlation analysis between NRS Score and CD56dim natural killer cell. **(H)** Correlation analysis between NRS Score and Eosinophil. NS indicates no significant difference.

**Figure 8:**
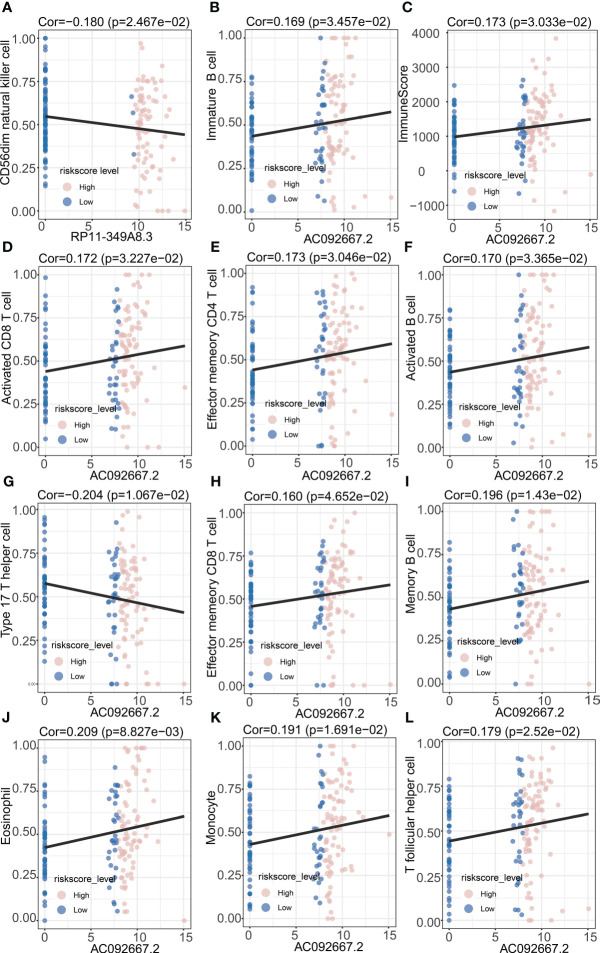
Correlation Analysis of prognosis-related lncRNAs and immune infiltrating cells. **(A)** Correlation analysis between RP11-349A8.3 and CD56dim natural killer cell. **(B)** Correlation analysis between AC092667.2 and Immature B cell. **(C)** Correlation analysis between AC092667.2 and ImmuneScore. **(D)** Correlation analysis between AC092667.2 and Activated CD8 T cell. **(E)** Correlation analysis between AC092667.2 and Effector memeory CD4 T cell. **(F)** Correlation analysis between AC092667.2 and Activated B cell. **(G)** Correlation analysis between AC092667.2 and Type 17 T helper cell. **(H)** Correlation analysis between AC092667.2 and Effector memeory CD8 T cell. **(I)** Correlation analysis between AC092667.2 and Memory B cell. **(J)** Correlation analysis between AC092667.2 and Eosinophil. **(K)** Correlation analysis between AC092667.2 and Monocyte. **(L)** Correlation analysis between AC092667.2 and T follicular helper cell.

In order to study the tumor-suppressive immunoregulatory pattern, we obtained the relative abundance of 28 infiltrating immune cells by Single-sample Gene Set Enrichment Analysis (ssGSEA) analysis (**Figure 7A**). However, we found that among the two groups of patients divided by the median NRS Score, there is no significant difference in the stromal score and immune scores, which makes us further explore the immune -infiltrating cells (**Figures 7B-D**). Pearson correlation analysis shows that, the abundance of immune cells with anti-tumor function (such as Activated CD4 T cell, Activated CD8 T cell, Central memory CD4 T cell, Central memory CD8 T cell) and tumor-suppressor immune cells (such as Regulatory T cell, Type2 T helper cell, CD56dim natural cell) was significantly positively correlated in microenvironment classified by risk score (**Figure 7E**). At the same time, we found a negative correlation between NRS Score and Type 17 T helper cell (**Figure 7F**), and a significantly positive correlation between NRS Score and CD56dim Natural Killer cell or eosinofile (**Figures 7G, H**). In addition, we found a negative correlation between RP11-349A8.3 and CD56dim Natural Killer Cell. AC092667.2 was positively correlated with the immunologic infiltrating cells such as Immaturity B cell, Activated CD8 T cell, Effector memeory CD4 T cell, Activated B cell, Effector memeory CD8 T cell, Memory B cell, Eosinophil, Monocyte and T follicular helper cell, there is a negative correlation with Type 17T helper cells (**Figures 8A, B, D-L**). At the same time, we found a significant positive correlation between AC092667.2 and ImmuneScore (**Figure 8C**). This suggests that NOX4-associated lncRNA plays an important role in the immune microenvironment of pancreatic cancer.

### 3.7 Immune microenvironment in patients with different immune states

**Figure 9:**
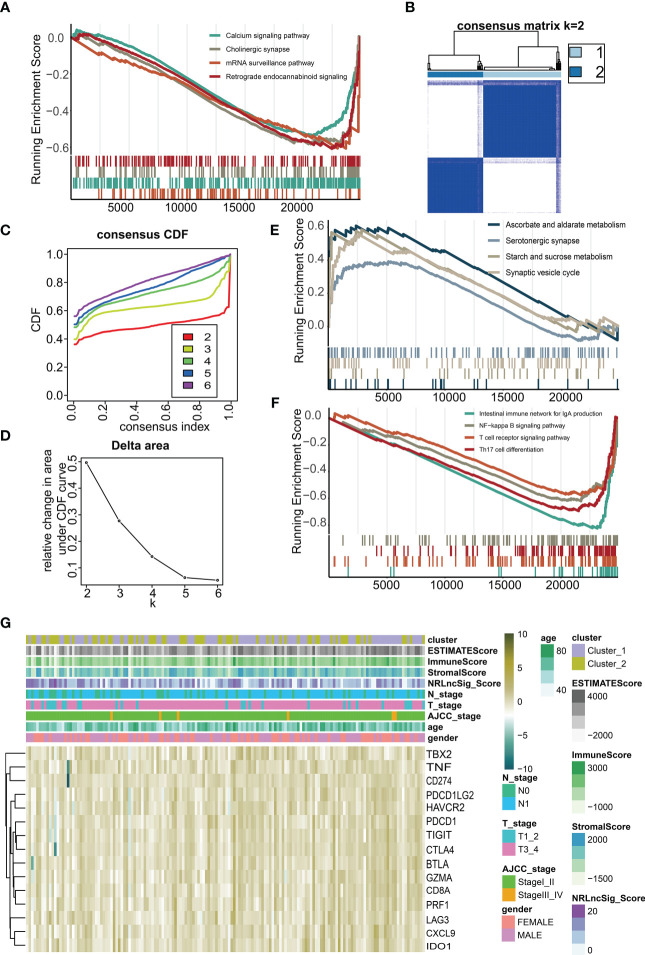
Immune microenvironment of different immune subtypes. **(A)** Gene set enrichment analysis (GSEA) for NRS Score. **(B)** Two immune subtypes were identified by unsupervised consensus clustering based on K-means. **(C)** Consensus values range from 0 to 1. **(D)** The corresponding relative change in area under the cumulative distribution function (CDF) curves. The range of k changed from 2 to 6, and the optimal k = 2. **(E, F)** Gene set enrichment analysis (GSEA) for immune subtypes. **(G)** Heatmap of the expression of immune checkpoints under different immune subtypes and immune scores.

We performed GSEA between high NRS Score and low NRS Score group, and found that pathways such as Calcium signaling pathway, Cholinergic synapse, mRNA surveillance pathway and Retrograde endocannabinoid signaling were enriched in low NRS Score group (**Figure 9A**). By unsupervised consensus clustering based on K-means, we identified two immune subtypes. (**Figures 9B-D**). We found that immune subtype cluster 2 were more concentrated in immune response-related pathways (**Figures 9E, F**), such as Intestinal immune network for IgA production, NF-kappa B signaling pathway, T cell signaling pathway and Th17 cell differentiation pathway. The Heatmap showed a higher level of expression of immune checkpoints (**Figure 9G**) in immune subtype cluster 1.

### 3.8 Drug sensitivity analysis

**Figure 10:**
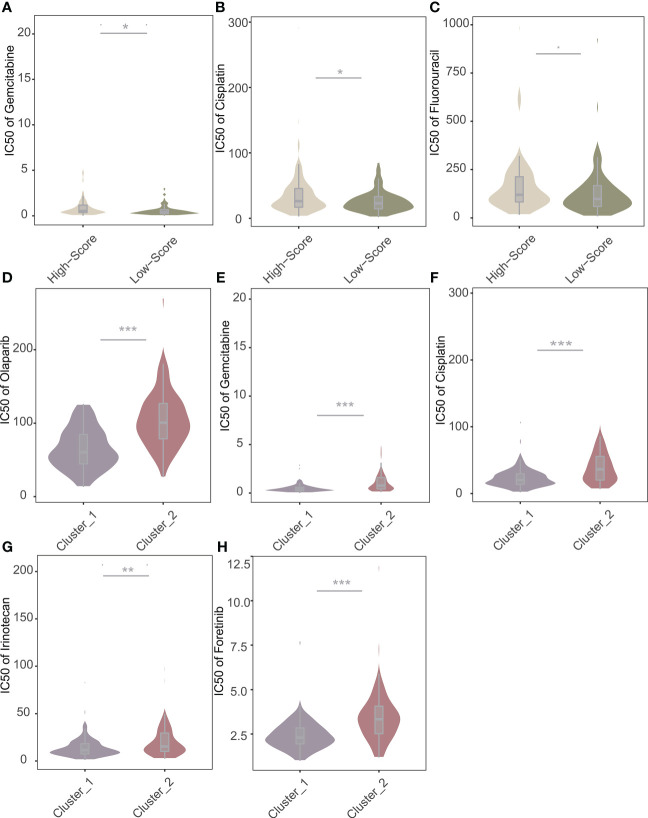
Predictive value of NRS Score and immune subtypes for therapeutic response of chemotherapeutic agents. **(A)** Comparisons of IC50 of Gemcitabine for chemotherapeutics and targeted therapy drugs between two score stratifications. **(B)** Comparisons of IC50 of Cisplatin for chemotherapeutics and targeted therapy drugs between two score stratifications. **(C)** Comparisons of IC50 of Fluorouracil for chemotherapeutics and targeted therapy drugs between two score stratifications. **(D)** Comparisons of IC50 of Olaparib for chemotherapeutics and targeted therapy drugs between two immune subtypes. **(E)** Comparisons of IC50 of Gemcitabine for chemotherapeutics and targeted therapy drugs between two immune subtypes. **(F)** Comparisons of IC50 of Cisplatin for chemotherapeutics and targeted therapy drugs between two immune subtypes. **(G)** Comparisons of IC50 of Irinotecan for chemotherapeutics and targeted therapy drugs between two immune subtypes. **(H)** Comparisons of IC50 of Foretinib for chemotherapeutics and targeted therapy drugs between two immune subtypes. *p < 0.05, **p < 0.01, ***p < 0.001.

Based on the “oncoPredict” algorithm, we calculated the IC50 of each sample to evaluate the predictive power of the NRS Score for clinical chemotherapy efficacy. W We analyzed 8 conventional chemotherapeutic agents (Olaparib, Paclitaxel, Gemcitabine, Cisplatin, Irinotecan, Oxaliplatin, Fluorouracil, Foretinib), commonly used for pancreatic cancer, predicting the IC50 of patients with different scores (**Figures 10A-C**). The results showed that the IC50 of Gemcitabine, Cisplatin and Fluorouracil were significantly different among patients grouped with NRS Score, and the IC50 of high score group were significantly higher than those of low score group. We predicted chemosensitivity in patients with different immune subtypes. Patients identified as immune subtype cluster 1 were more sensitive to Gemcitabine, Cisplatin, Irinotecan, Foretinib and Olaparib than patients identified as immune subtype cluster 2 (**Figures 10D-H**). This indicates that the related lncRNAs score based on NOX4 has a new explanation for the mechanism of drug resistance in pancreatic cancer and can well predict the chemotherapy response.

## 4 Discussion

Recent results based on pancreatic cancer have not significantly improved the overall prognosis of patients. At the same time, treatment targeting tumor stroma or hypoxic microenvironment has not achieved ideal results (24). Molecular heterogeneity, peritumoral stroma and inflammatory factors in pancreatic cancer contribute to the signal transduction of the whole tumor microenvironment very complex, and drug targeting is very difficult. Uncontrollable genetic mutations and the immune microenvironment of patients with pancreatic cancer has a unique immunosuppressive effect, which can promote the development of tumor (25). In patients with pancreatic cancer, the quality of neoantigens is associated with overall survival, suggesting that adaptive immunity is associated with pancreatic cancer (11). The early symptoms of pancreatic cancer are not obvious and screening is difficult. At the beginning of the study, we expected to find the relationship between the immune microenvironment and each clinical stage, and to discuss the tumor infiltration in the individual. More detailed stratification of the patient may be required to ensure individualized and accurate treatment. In recent years, lncRNAs have been found to play an important role in the immune microenvironment of pancreatic cancer, and the corresponding treatment mechanism also urgently needs to discuss. This study evaluated the prognosis of patients with pancreatic cancer based on the validated pancreatic cancer markers (NOX4) and NOX4-related lncRNA under subgroups of clinical indicators and to explore their relationship with the immune microenvironment.

NOX4, a member of the NOXs family that produces reactive oxygen species, has been shown to be associated with the occurrence and development of many cancers. It has been demonstrated that the expression of NOX4 is increased in NSCLC cells and further promotes the development of NSCLC by promoting glycolysis through the ROS/PI3K pathway (16, 26). NOX4 is essential for maintaining the phenotype of immunosuppressive tumor-associated fibroblasts (CAF). Molecular studies based on the cellular level and tumor models in animals have demonstrated that NOX4 plays an important role in tumor metabolism and microenvironment regulation. However, the reality is that the *in vivo* environment and disease progression in pancreatic cancer patients is significantly off the expectation. More ethical research results for different individuals need to be obtained before implementing clinical targeted therapies. To address this research gap, a large number of pathological samples from pancreatic cancer patients were collected for analysis. According to the validation of a public database, we found that NOX4 was highly expressed in tumor tissues, and high expression of NOX4 predicts poor prognosis in patients with pancreatic cancer. Meanwhile, At the same time, in combination with clinical case analysis and immunohistochemical analysis can be seen due to the clinical characteristics of different, the expression of NOX4 changes. We quantified and graded the positive rate of NOX4 in various patient tissue and found that a group of patients with low NOX4 positive scores had the best prognosis. We identified 166 DEGs in a NOX4-graded gene profile. The inhibition of NOX4 can enhance the effect of immunotherapy by antagonizing the rejection of CD8+ T cells mediated by CAF (27). Bauer Km et al. found that overexpression of NOX4 in left-sided colon cancer predicted poor survival. Meanwhile, the study found that the 5-year survival rate was 0.51(95% CI 0.37 ~ 0.70) in patients with left colon tumors with high expression of NOX4, the 5-year survival rate for patients with low NOX4 expression was 0.89(95%CI 0.80 ~ 0.99) (28). KEGG analysis showed that most of the DEGs were enriched into pathways such as Synaptic vescycle, panic secretion and Protein digestion and absorption. The results of these enrichment analyses do not seem to provide a better understanding of the mechanism of NOX4 in the tumor microenvironment. Because the difference in the expression of the same gene is small in patients, the relative differential expression of mRNA is not a major factor that leads to the major role of NOX4 in the tumor immune microenvironment. We turn our attention to the associated lncRNA, these potential regulatory molecules that may be key to helping NOX4 play a role in the immune system.

In recent years, more and more tumor-related lncRNAs have been found and successfully annotated as potential cancer therapeutic targets. MACC1-AS1 has been identified as an overexpression of lncRNA in pancreatic cancer. Inhibition of MACC 1-AS1 gene can inhibit the proliferation and metastasis of pancreatic cancer cells. High expression of MACC1-AS1 predicts poor prognosis (29). In addition, the overexpression of LINC00462 enhances cell migration and invasion by enhancing epithelial-mesenchymal transition (EMT) and accelerating the growth of pancreatic cancer cell (30). In pancreatic cancer cells, lncRNA MEG3 was found to be down-regulated. The expression of MEG3 has been shown to be negatively correlated with tumor metastasis and vascular invasion in pancreatic cancer (31) Similarly, lncRNA Gas5 was found to inhibit metastasis of pancreatic cancer by regulating the miR-32-5p/PTEN pathway (32). In this study, 986 lncRNAs with high correlation to NOX4 were selected by Spearman’s correlation analysis, and 12 lncRNAs were selected as feature vectors by the Least Absolute Shrinkage and Selection Operator (LASSO). Finally, by performing univariate and multivariate COX regression analysis, two NOX4-related lncRNAs were identified as having significant prognostic significance (RP11-349A8.3, AC092667.2). We calculated the NRS Score for each patient and divided the patients into high-score and low-score groups based on the median. The Kaplan-Meier survival curve showed that patients with higher scores had longer survival times. The ROC curve and DCA curve can prove that the model has good prediction performance. And NRS Score was more reliable in predicting the prognosis of patients than either of the prognostic lncRNAs and clinical variables. Although lncRNAs have been shown to be involved in the activation of NOX4-related signaling axis in several diseases, it is rare to study about the relationship between NOX4 and lncRNAs in tumors. Based on the implication of the above-mentioned results, our research result on the role played by NOX4-related lncRNAs in pancreatic cancer and the intervention on the immune microenvironment provides a foundation for the subsequent mechanistic exploration.

We found a significantly positive correlation between the abundance of immune cells with anti-tumor function (such as Activated CD4 T cell, Activated CD8 T cell, Central memory CD4 T cell, Central memory CD8 T cell) and tumor-suppressor immune cells (such as Regulatory T cell, Type2 T helper cell, CD56dim natural cell) in the high-and low-score groups. At the same time, we found that NRS Score had a negative correlation with Type 17 T helper cell or CD56dim Natural Killer cell, and a positive correlation with Eosinophil, these results suggest that NRS Score can be used as an effective index to evaluate immune infiltrating cells in pancreatic tumor microenvironment. Th17 cells have been shown to be a key regulator of autoimmunity. Studies based on melanoma models *in vitro* have shown that metastatic polarized Th17 cells can induce tumor degeneration and destruction, and reduce the number of tumors (33). Th17 cells can stimulate CD8 + CTL response through IL-2 and pMHC I, and stimulate the expression of CCL2 and CCL20 in tumor microenvironment to promote the recruitment of various inflammatory leukocytes (DCs, CD4 + and CD8+ T cells) (34). CD56dim CD16-NK cells have been shown to be a unique non-cytolytic subset in melanoma and may predict better prognosis in patients with melanoma (35). Eosinophil has indirect tumor promoting activity in TME by regulating the composition or activity of immune cells, secreting growth factors and matrix metalloproteinase, and promoting angiogenesis. Tumor-derived thymic stromal lymphopoietin (TSLP) can activate eosinophil to increase the expression of IL-4, IL-5, IL-10 and IL-13, and promote the proliferation of cervical cancer cells (36). Kratochvill et al. demonstrated that eosinophil can promote tumor growth by secreting IL-4 and IL-13, and may induce macrophage polarization in the tumor microenvironment (37). At the same time, eosinophil can promote the formation of inhibitory tumor microenvironment by producing indoleamine 2,3-dioxygenase (IDO) and induce tumor resistance (38). We also studied the relationship between these two lncRNAs and immune infiltrating cells. RP11-349A8.3 was significantly correlated with immaturity B cell and CD56dim Natural Killer cell. AC092667.2 showed significant correlation with Activated CD8 T cell, Effector memeory CD8 T cell, Effector memeory CD4 T cell, Type 17 T helper cell and T follicular helper cell, the positive correlation between AC092667.2 and ImmuneScore suggests that these two lncRNAs have not been studied may play an important role in tumor immune microenvironment. Pancreatic cancer is often described as a “cold tumor” with a relative lack of CD8+ T cells and a marked heterogeneity of T cell infiltration. Now, the density of the tumor’s effector T cells has also been shown to correlate with improved prognosis (39). We reviewed existing basic research, and no one seems to be looking at these two lncRNAs (RP11-349A8.3, AC092667.2), indicating a new breakthrough in the exploration of immune cell infiltration and immune mechanisms in pancreatic cancer.

Through ssGSEA, we obtained the expression of 28 immune cells per patient. All patients were divided into two immune subtypes by unsupervised consensus clustering based on K-means. We found that immune subtype cluster 1 had higher levels of infiltrating cells, and the GSEA results showed that several immune-related pathways were enriched in immune subtype cluster 1. Studies by Qingcai Meng et al. have demonstrated that NF-κB, as a target of miR-146a-5p, participates in the regulation of pancreatic resistant to systemic chemotherapy (40). While Th17 and T regulatory cells can create a fibrotic, inflammatory and immunosuppressive environment, and significantly inhibit the number of cytotoxic T cells. Th17 cells can acquire Th1-like functions and characteristics after being activated, which plays a role in enhancing autoimmunity and anti-tumor immunity (41). The importance of T cell overactivation for disability and depletion has been demonstrated by some T cell receptor pathways, such as nuclear factor of activated T-cells (NFAT) and sprouty homolog 2 (SPRY2). The expression of inhibitory T cell receptor is related to TCR signal and indicates the activation of negative regulatory pathway (42). TCR activation pathways such as phosphatidylinositol 3-kinase (PI3K), protein kinase Akt and the signaling molecule RAS have been shown to cross-link with depletion and PD-1 (43).

NRS Score is a reliable predictor of the efficacy of chemotherapy. Further expanding the study of lncRNAs interacting with some mature tumor markers is of great significance for targeted therapy. It is reasonable to suspect that NOX4-related lncRNAs play a role in the development of chemoresistance. Systemic therapy based on chemotherapy is the main clinical strategy for pancreatic cancer. Gemcitabine is widely used as a first line treatment for advanced pancreatic cancer. With 5-fluorouracil(5-FU) as the clinical control group, the median overall survival (5.65 to 4.41 months) and one-year survival (18% to 2%) were significantly increased in Gemcitabine-treated group (44). The development of chemoresistance may be related to the activation and inactivation of drug transporters, related enzymes and their targets. The transport, absorption and metabolism of Gemcitabine are regulated by a variety of enzymes, and the formation of drug resistance is influenced by tumor microenvironment, epithelial-mesenchymal transformation, microRNA and other factors (45). Our study of the sensitivity of patients with different immune states to chemotherapy further suggests the use of immunotherapy as adjuvant chemotherapy, in combination with chemotherapy, radiotherapy, and antiangiogenic agents, can significantly improve the clinical efficacy of treatment.

And the results of this study suggest that NOX4-related lncRNAs seem to exhibit more obvious results than NOX4 in pathological analysis and individual diagnosis for pancreatic cancer patients in different states. lncRNAs have been proven as ideal markers. The use of techniques such as liquid biopsy in clinical diagnosis makes it possible to validate the diagnosis of pancreatic cancer based on NOX4-related lncRNAs. Our study provides a foundation for subsequent exploration and a control for subsequent *in vivo* experiments involving molecular mechanisms.

However, the study has some limitations. Firstly, immunohistochemical analysis of a large number of tissue samples inevitably has deviation in tissue collection and batch effect in experimental operation. Secondly, this study only analyzed the clinical data of patients collected by surgery and RNA-seq data from TCGA database. We screened several datasets from GEO database and ICGC database, unfortunately, due to the lack of annotation or the instability of genetic abundance, no suitable data were found for further verification. We still need the data for external validation to evaluate the applicability and reliability of the relevant models. Finally, more *in vitro* and *in vivo* studies are needed to demonstrate the mechanism of NOX4-related lncRNAs in pancreatic cancer.

As a difficult early diagnosis and poor prognosis cancer, pancreatic cancer is full of difficult in clinical treatment. This study shows that NOX4 are suitable biomarkers for pancreatic cancer, and the lncRNAs influenced by NOX4 is closely related to the prognosis of patients. Thus, the NRS Score can independently predict the prognosis of patients with pancreatic cancer and explain the effect of immunotherapy and chemotherapy resistance.

## Data availability statement

The original contributions presented in the study are included in the article/supplementary materials. Further inquiries can be directed to the corresponding author.

## Author contributions

XZ, XM, and LF conceived the original idea of this manuscript and designed the study. YH and YP wrote the manuscript and figures. XZ, LY, and LL were responsible for data collection and data analysis. XM provided support for algorithmic principles and computer equipment. XZ and LF reviewed the finished manuscript and supervised throughout the process. All authors contributed to the article and approved the submitted version.
